# Atypical clinical features of mpox (monkeypox): a diagnostic challenge - Reply^⋆^

**DOI:** 10.1016/j.abd.2023.04.002

**Published:** 2023-05-23

**Authors:** Elena Lucía Pinto-Pulido, Miriam Fernández-Parrado, Francisco José Rodríguez-Cuadrado

**Affiliations:** a Department of Dermatology, Hospital Universitario Príncipe de Asturias, Universidad de Alcalá, Madrid, Spain; b Department of Dermatology, Hospital Universitario de Navarra, Pamplona, Spain; c Department of Dermatology, Hospital Universitario Puerta de Hierro, Madrid, Spain

Dear Editor,

We recently published a mpox concise review focused on the new features of dermatological lesions that were observed during the 2022 outbreak.^1^ In response to this publication, Kleebayoon and Wiwanitkit^2^ submitted an interesting letter pointing out the existence of mpox cases where diagnosis is challenging due to atypical or initially absent skin lesions.

We agree that a significant percentage of patients do not initially present mucocutaneous lesions. Large 2022 case series reported 36%‒61.5% of patients develop unspecific systemic prodromic manifestations (fever, malaise, lymphadenopathy, headache).^1^ Accurate diagnosis prior to the presence of mucocutaneous lesions is difficult. However, it is noteworthy that in this outbreak the absence of systemic symptoms or their appearance after the cutaneous manifestations was more frequent compared to previous outbreaks of classical mpox.^3^

Although typical skin lesions consist of pseudopustules which may be followed by a second exanthematous phase, other more infrequent skin lesions have been described. For example, some patients develop a finger whitlow. In a 2022 study, mpox whitlows, together with mucosa and single skin lesions led to clinical misdiagnosis.^3^

Mucosal involvement is not uncommon, but symptoms are unspecific, mimicking other possible diagnoses. Oropharyngeal manifestations include oral and tonsillar ulcers, epiglottitis, and pharyngitis, leading to odynophagia and dysphagia. The anorectal mucosa can be affected with ulcerations or proctitis, which produce symptoms such as pain, bleeding, tenesmus, or diarrhea. Conjunctival mucosa involvement is more infrequent but can lead to keratitis and vision loss.^4^

As Kleebayoon and Wiwanitkit^2^ claim, Polymerase Chain Reaction (PCR) testing may present false positives and negatives. Nevertheless, these limitations are inherent to any diagnostic test. We, therefore, agree that sampling and analysis should be repeated in doubtful cases. Atypical cases may even require a skin biopsy to make an accurate diagnosis.^5^ However, to date, the PCR test is the main confirmatory diagnostic method. Skin lesions have the highest diagnostic yield (sensitivity 91%–100%) but other samples (oral, nasopharyngeal, or rectal swab) can also be collected and assess laboratory diagnosis in cases without skin lesions. On the contrary, there is no clear evidence about the diagnostic reliability of blood, urine, and feces PCR testing. Mpox rapid antigen tests have also been developed. Their practical utility is unclear because they have lower sensitivity than PCR tests. However, they could be helpful if PCR testing is not available.^6^

In order to make mpox diagnosis easier for clinicians, several artificial intelligence tools have been developed. They recognize images of mpox typical skin lesions, differentiating them from images of other diseases. Most of them achieved high accuracy. Notwithstanding, they have not been tested in real life and some privacy issues remain to be solved.^7^

## Financial support

None declared.

## Authors' contributions

Elena Lucía Pinto-Pulido: Approval of the final version of the manuscript; critical literature review; data collection, analysis, and interpretation; effective participation in research orientation; preparation and writing of the manuscript; study conception and planning.

Miriam Fernández-Parrado: Approval of the final version of the manuscript; critical literature review; data collection, analysis, and interpretation; effective participation in research orientation; manuscript critical review; study conception and planning.

Francisco José Rodríguez-Cuadrado: Approval of the final version of the manuscript; critical literature review; data collection, analysis, and interpretation; effective participation in research orientation; manuscript critical review; study conception and planning.

## Conflicts of interest

None declared.
